# Detection and phenotyping of extracellular vesicles by size exclusion chromatography coupled with on-line fluorescence detection

**DOI:** 10.1038/s41598-019-56375-1

**Published:** 2019-12-27

**Authors:** Diána Kitka, Judith Mihály, Jean-Luc Fraikin, Tamás Beke-Somfai, Zoltán Varga

**Affiliations:** 1 Biological Nanochemistry Research Group, Institute of Materials and Environmental Chemistry, Research Centre for Natural Sciences, Budapest, Hungary; 2 Spectradyne LLC, Torrance, CA USA

**Keywords:** Biochemistry, Biological techniques, Biomarkers

## Abstract

New methods for quantifying extracellular vesicles (EVs) in complex biofluids are critically needed. We report the development of a new technology combining size exclusion chromatography (SEC), a commonly used EV purification technique, with fluorescence detection of specifically labelled EVs. The resulting platform, Flu-SEC, demonstrates a linear response to concentration of specific EVs and could form the basis of a system with phenotyping capability. Flu-SEC was validated using red blood cell derived EVs (REVs), which provide an ideal EV model with monodisperse size distribution and high EV concentration. Microfluidic Resistive Pulse Sensing (MRPS) was used to accurately determine the size distribution and concentration of REVs. Anti-CD235a antibody, specific to glycophorin A, and the more general wheat germ agglutinin (WGA), were selected to label REVs. The results show the quantitative power of Flu-SEC: a highly linear fluorescence response over a wide range of concentrations. Moreover, the Flu-SEC technique reports the ratio of EV-bound and free-antibody molecules, an important metric for determining optimal labelling conditions for other applications. Flu-SEC represents an orthogonal tool to single-particle fluorescent methods such as flow cytometry and fluorescent NTA, for the quantification and phenotyping of EVs.

## Introduction

With diverse physiological and pathological roles, extracellular vesicles (EVs) offer enormous potential as biomarkers and therapeutics^1–6^. However, the isolation of EVs from complex body fluids and the subsequent characterization of the purified EVs are still technically challenging^7–10^. Recently, size exclusion chromatography (SEC, also called gel permeation chromatography) has been broadly adopted as a convenient and accessible technique for the purification of EVs^11–16^. SEC is a size-based method that effectively separates nanoscale particles such as EVs from smaller molecules and impurities. Currently, the most common SEC-based EV-purification procedures employ manual or centrifugal columns. During SEC purification, discrete fractions are collected and subsequently analysed for particle size and composition. Liquid chromatography systems with on-line UV-visible absorption, fluorescence, or light scattering detectors are rarely used in EV research, though fluorescence detection in particular would be especially valuable because it enables specific measurements of fluorescently labelled EVs. Labelling with fluorescent antibodies is widely used for quantifying EVs by flow cytometry^17,18^, immunoassays^19^ and recently with nanoparticle tracking analysis (NTA)^20,21^, but has not yet been tested in conjunction with SEC.

In this paper we report the development and validation of fluorescence SEC (Flu-SEC) for analysis of EVs in complex media using EV-specific labels. While the combination of fluorescence detection and SEC for the characterization of EVs has been described to some extend before^22,23^, prior studies have been limited to non-specific membrane dyes that have limited applicability for analysing EVs in complex body fluids. Red blood cell- (RBC-) derived EVs (REVs) were chosen to validate Flu-SEC, because REVs have a monodisperse size distribution from a single-cell origin and can be isolated in high concentration without other contaminating biological nanoparticles. We have labelled REVs with a fluorochrome conjugated antibody (PE-antiCD235a) against glycophorin A, a characteristic membrane protein of RBCs, and with a more general marker that binds to glycoproteins associated with the membrane of EVs (wheat germ agglutinin, WGA). By combining Flu-SEC with microfluidic resistive pulse sensing (MRPS), we were able to relate the measured fluorescence intensity to the number concentration of EVs. This initial validation of the Flu-SEC technique paves the way for Flu-SEC-based phenotyping of EVs in complex body fluids.

## Results

### Characterization of REVs

**Figure 1:**
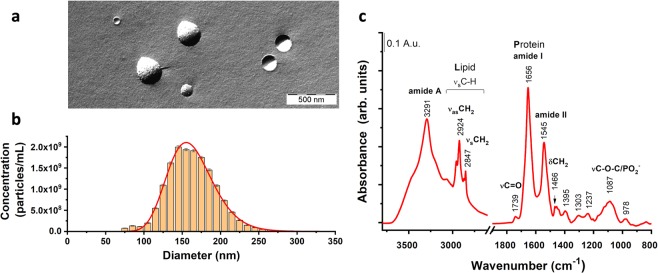
Characterization of red blood cell derived extracellular vesicles (REVs). Freeze-fracture combined transmission electron microscopy (FF-TEM) was used to visualize the morphology of REVs (**a**), size distribution and concentration of REVs was measured by microfluidic resistive pulse sensing (MRPS, **b**), and Fourier-transform infrared spectroscopy (FTIR) was used to characterize the protein and lipid content of the vesicles (**c**).

Morphological characterization of REVs was performed by freeze-fracture combined transmission electron microscopy (FF-TEM), which preserves the native structure of EVs. REVs were found to have spherical morphology with a typical diameter in the range of 100 nm to 300 nm (Fig. 1a).

The quantitative size distribution of the REV sample was measured using MRPS (Fig. 1b). The REV size distribution is monodisperse and is well fit by a log-normal distribution with mean diameter 163.5 ± 0.7 nm and standard deviation 30.6 ± 0.6 nm (adj. R-Square = 0.982). The concentration of REVs measured by MRPS was (3.140 ± 0.002) · 10^11^ particles/mL over the size range from 65 nm to 400 nm. This value was used for the dilution series during the Flu-SEC measurements.

The overall lipid and protein composition of the REVs was characterised using FTIR spectroscopy. The infrared absorption spectrum is shown in Fig. 1c. The characteristic bands for proteins at 3291, 1656 and 1545 cm^−1^, corresponding to amide A, amide I and amide II vibrations of the peptide backbone, as well as the asymmetric and symmetric methylene stretching of acyl chains at 2918 cm^−1^ and 2850 cm^−1^, respectively and the glycerol carbonyl stretching at 1739 cm^−1^ of the phospholipids show the typical spectral features of EVs. The shape and peak position of the amide I band indicate that helical structures dominate the protein content of REVs, consistent with the high haemoglobin content of REVs. The integrated areas of amide I band and that of the C-H stretching (from 3040 to 2800 cm^−1^ wavenumber region) bands were used to calculate the spectroscopic protein-to-lipid ratio, which is characteristic to the purity and type of EVs^24^. We obtained 1.3 ± 0.1 spectroscopic protein-to-lipid ratio for the REV sample used in this study, which is in good agreement with previous observations. The cellular origin of REVs was directly verified by fluorescence antibody labelling with PE-CD235a as discussed in detail in the next section.

### Flu-SEC measurements of REVs

**Figure 2:**
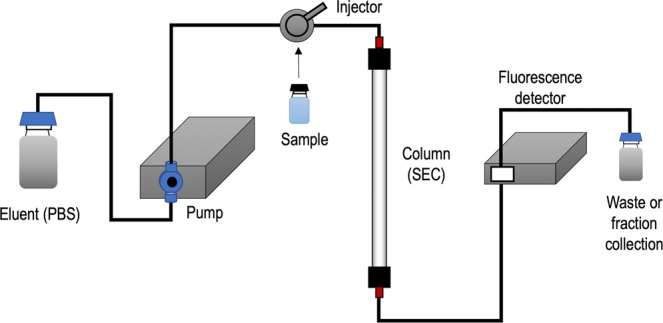
Schematic setup of the Flu-SEC measurements. In this system, the continuous eluent flow is provided by an HPLC pump, and the fluorescently labelled sample is loaded in the system via an injector. The sample passing through the column is analysed with an on-line fluorescence detector directly connected to the column outlet and controlled by the software of the liquid chromatography system.

**Figure 3:**
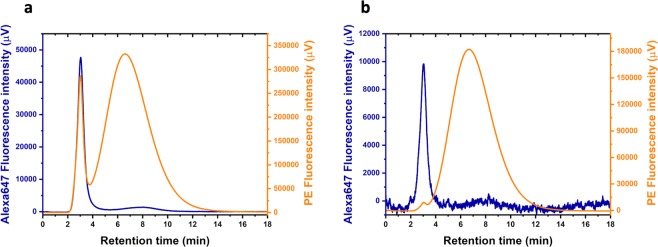
Flu-SEC chromatograms of REV samples fluorescently labelled at High (**a**) and Low (**b**) vesicle concentrations. EVs are eluted at 3 min retention time, while free PE-CD235a and Alexa647-WGA are eluted at 6.6 min and 7.9 min, respectively.

The schematic setup of the Flu-SEC measurements is shown in Fig. 2. REV samples were prepared at High and Low concentrations, labelled with two fluorophores, Alexa647-WGA and PE-CD235a as described in Table 1, and analysed by Flu-SEC. Chromatograms for REV High and REV Low are shown in Fig. 3a,b respectively.

**Table 1:** EV and fluorescent label concentrations used in the Flu-SEC experiments.

	Concentration (vesicles · mL^−1^)	[Alexa647-WGA] (μg/mL)	[PE-CD235a] (μg/mL)
REV High	3.14 · 10^11^	8	0.8
REV Low	6.28 · 10^9^	2	0.2

EVs are eluted in the first sharp peak at 3 min retention time, which corresponds to 1.5 mL elution volume. For Alexa647-WGA labelled samples, a second peak corresponding to the free label is detected with much lower intensity at 7.9 min retention time. The area under the curve (AUC) values for each peak indicate that 87.8% and 79.7% of the total Alexa647-WGA is bound to EVs in the REV High and REV Low samples respectively. For PE-CD235a labelled samples, the free label is detected at 6.6 min retention time and a more significant difference is observed: While 12.7% of the total label is bound to EVs in the REV High sample, only 0.8% is bound in the REV Low sample, suggesting that Alexa647-WGA has a higher equilibrium binding constant than the PE-CD235a antibody.

**Figure 4:**
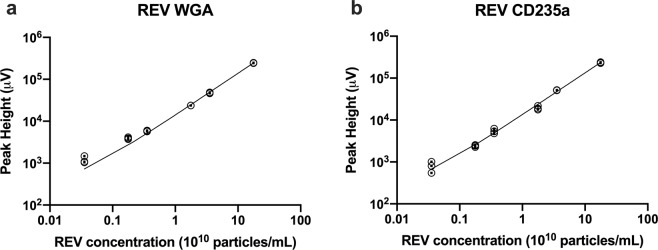
Dependence of the peak height of the EV fluorescence peak from the number concentration of REVs using Alexa647-WGA (**a**) and PE-CD235a (**b**) for labelling at 0.2 μg/mL concentrations. Concentration series were measured between 3.54 · 10^8^ mL^−1^ and 1.77 · 10^11^ mL^−1^. Individual replicates (N = 3) are shown for each concentration with symbols, and the best fitting linear functions are shown with solid lines on a log-log plot.

Next, the linearity of the relationship between the first fluorescence peak and the number concentration of REVs was examined. Figure 4 shows the dependence of the fluorescence peak height of the EV-peak from the REV number concentration measured from 3.54 · 10^8^ mL^−1^ to 1.77 · 10^11^ mL^−1^ REV number concentration with 0.2 μg/mL label concentration for both Alexa647-WGA (Fig. 4a) and PE-CD235a (Fig. 4b). Good linearity with adjusted R-Square values of above 0.99 were obtained for the studied concentration ranges. Calculating the limit of detection and the limit of quantitation (LOD = 3S_a_/b and LOQ = 10S_a_/b, respectively, where b is the slope of the fitted linear function, and S_a_ is the standard deviation of the y-intercept) from the linear regressions we obtained LOD values of 1.03· 10^9^ mL^−1^ and 2.2 · 10^9^ mL^−1^ for Alexa647-WGA and PE-CD235a labelled REVs, respectively, and LOQ values of 3.4· 10^9^ mL^−1^ and 7.3 · 10^9^ mL^−1^ for Alexa647-WGA and PE-CD235a labelled REVs, respectively.

## Discussion

According to TEM, MRPS and FTIR measurements, REV samples used in this study have spherical morphology, a monodisperse log-normal size distribution with mean diameter near 163 nm, and lipid and protein compositions characteristic of EVs. The cellular origin of REVs is verified by the Flu-SEC investigations, which clearly indicated that these EVs are positive for the RBC-marker CD235a. Based on these properties, REVs are ideal model EVs for validating Flu-SEC for the quantification of EVs.

The fluorescence chromatograms of the REV samples labelled with PE-CD235a and with Alexa647-WGA (Fig. 3) show the typical features of the separation of EVs from soluble proteins with SEC. The position of the EV peak confirms that REVs are completely excluded from the pores of the Sepharose CL-2B gel, which has a size exclusion limit of 40 × 10^6^ Da for globular proteins (corresponding to a sphere with a diameter of 45 nm assuming density of 1.37 g/cm^3^): The total volume of the column is V_T_≈4 mL and EVs elute at 1.5 mL, which corresponds to the void volume (V_0_≈V_T_/3), and the EV size measured by MRPS (163 nm) exceeds the equivalent particle size exclusion limit. In contrast, the labelling protein Alexa647-WGA has an approximate molecular weight of 38 to 40 kDa including fluorophores, which falls below the lower separation limit of 70 kDa for Sepharose CL-2B. Correspondingly, the elution volume of the peak of free Alexa647-WGA molecules (7.9 min × 0.5 mL/min = 3.95 mL) equals the total volume of the column. Free PE-CD235a is eluted at 6.6 min × 0.5 mL/min = 3.3 mL elution volume, consistent with the much higher molecular weight of PE-CD235a (approx. 390 kDa). The significant breadth of the free label peaks relative to the EV-peak is expected as a result of diffusion during the course of chromatography^25^. The mean squared displacement of a particle in an amount of time t is proportional to D × t, where the diffusion constant, D, is inversely proportional to particle diameter according to the Stokes-Einstein equation. The peak broadening of the free label particles is therefore much greater than that of EVs, since the labels are smaller and are retained for longer times than the EVs.

As the free label peak increases in breadth and intensity, it begins to overlap with the EV peak in the chromatogram—ultimately it may interfere with the quantification of EVs and impact the low concentration limit of detection. The label concentration should therefore be chosen in accordance with the concentration of EVs as demonstrated in this study: With this strategy the linear range for quantification of EVs in our experiments spans over two orders of magnitude ranging from 10^9^ mL^−1^ to 10^11^ mL^−1^. The LOD and LOQ values, however, strongly depends on the type of the label, the brightness of the fluorochrome and the sensitivity of the fluorescence detector.

The significant differences observed in relative peak ratios between REV High and REV Low for each label is related to the equilibrium constant of the antigen – antibody or the lectin – sugar binding, and the binding site density on the surface of EVs. The near equality of the peak AUC ratios for Alexa647-WGA labelled REV High and REV Low indicates a strong binding of this label to REVs at the concentrations tested. For PE-CD235a however, the ratio of the free-label peak AUC to the EV peak AUC increases with decreasing EV concentration, indicating a weaker binding of PE-CD235a to EVs. These observations can be explained by the fact that WGA binds to all glycoproteins bearing sialic acid or N-acetyl-glucosamin units, whereas PE-CD235a binds to Glycophorin A only.

Flu-SEC is a powerful method for the quantitative measurement of fluorescently labeled EVs in diverse applications. Because LC is an established technique in clinical chemistry, Flu-SEC can practically be translated into clinical applications. The method can also be performed without an HPLC system, making it a technique that is broadly accessible to labs with varying resources: A wide range of FPLC (fast protein liquid chromatography) systems, such us the ÄKTA (GE Healthcare) or NGC (BioRad Laboratories) could be used in lieu of HPLC. The separation of the free fluorescent label from the labelled EVs could also be performed with appropriate manual SEC columns, and subsequent analysis performed with a fluorescence spectrometer or a plate reader with fluorescence detector. Flu-SEC also offers significant benefits to other fluorescence-based EV quantification techniques. Because it measures the concentration of free label in addition to the EV-bound label, Flu-SEC represents a tool for optimizing the label concentration for single-particle fluorescence technologies such as flow cytometry or fluorescent NTA that suffer reduced sensitivity (increased noise background) in the presence of excess free label.

## Conclusion

The Flu-SEC method described in this technical report is a powerful technique for quantifying specific EVs using fluorescent labels and represents an alternative to single-particle fluorescent methods such as flow cytometry and fluorescent NTA. Flu-SEC is an ensemble technique, meaning the fluorescence signal detected in the EV-peak originates from an ensemble of EVs. Therefore, even weakly-labelled EVs contribute to the Flu-SEC signal, providing an opportunity for improved concentration accuracy compared to flow cytometry, for which only those EVs that contain sufficient copies of the label are detected. Flu-SEC also provides a direct method for quantifying the EV labelling efficiency, an important metric for other single-particle fluorescence detection methods. Coupled with MRPS as a reference for EV quantification, Flu-SEC delivered insights about the binding kinetics of the fluorescent labels and showed excellent linearity of response over a wide range of EV concentrations.

## Methods

### Isolation of red blood cell derived EVs (REVs)

EDTA-anticoagulated blood was collected from healthy volunteers (3 × 6 mL) with informed consent by venepuncture without a tourniquet through a 21-gauge needle by use of a vacutainer system (Geiner Bio-One). During the entire investigation period, we followed the guidelines and regulations of the Helsinki Declaration in 1975, and the use of human blood samples was approved by the Scientific and Research Ethics Committee of the Hungarian Medical Research Council (ETT TUKEB 6449-2/2015). Cellular components were sedimented from whole blood by centrifugation at 2500 × g for 10 min (Nüve NF 800 R centrifuge, swing-out rotor). Plasma and the white blood cell containing buffy coat were removed and RBCs were suspended in equal volume of saline solution (0.9%) and washed three times at 2500 × g 10 min at 4 °C. After washing, RBCs were diluted with equal volume of phosphate buffered saline pH 7.4 (PBS, Sigma-Aldrich, Hungary) and were kept at 4 °C for 7 days. At the end of the incubation period, the RBCs were removed by centrifugation at 1500 × g for 10 min followed by another centrifugation step at 2850 × g for 30 min. The RBC-free supernatant was aliquoted into 2 mL Eppendorf tubes and pelleted at 16 000 × g for 30 min (Eppendorf 5415 R, F45-24-11 rotor, Austria). Each pellet was resuspended in 100 μl PBS. The REV sample was further purified with SEC using a 3.5 mL gravity column filled with Sepharose CL-2B gel (GE Healthcare, Sweden). 100 μl REV sample was pipetted onto the column which was followed by 900 μl PBS, while the flow through was discarded. Next, the purified REVs were eluted with 1 mL PBS and collected. Purified samples were stored at 4 °C and were used within 48 hours after isolation.

### Freeze-fracture combined transmission electron microscopy (FF-TEM)

Freeze-fracture combined transmission electron microscopy (FF-TEM) was used to characterize the morphology of REVs^26^ because the fast rate of cooling during freezing preserves the native structure of the hydrated EVs. The REV sample was mixed with glycerol (Sigma-Aldrich, Hungary) which is used as cryoprotectant at 3:1 sample-to-glycerol volume ratio. Approx. 2 μL vesicle sample was pipetted onto a gold sample holder and frozen by placing it immediately into partially solidified Freon for 20 seconds. Fracturing was performed at −100 °C in a Balzers freeze-fracture device (Balzers BAF 400D, Balzers AG, Liechtenstein). The replicas of the fractured surfaces were made by platinum-carbon evaporation and then cleaned with a water solution of surfactant and washed with distilled water. The platinum-carbon replicas were placed on 200 mesh copper grids and examined in a MORGAGNI 268D (FEI, The Netherlands) transmission electron microscope.

### Microfluidic resistive pulse sensing (MRPS)

Microfluidic resistive pulse sensing (MRPS) is the nanoscale implementation of the coulter principle in a microfluidic cartridge^27–30^. MRPS measurements were performed with a nCS1 instrument (Spectradyne LLC, USA). The samples were diluted 20-fold with bovine serum albumin (BSA, Sigma-Aldrich, Hungary) solution at 1 mg/mL in PBS buffer (Sigma-Aldrich, Hungary), filtered through a VivaSpin 500, 100 kDa MWCO membrane filter (Sartorius, Germany) according to the manufacturer’s instructions. All measurements were performed using factory calibrated TS-400 cartridges with a measurement range from 65 nm to 400 nm.

### Fourier-transform infrared spectroscopy (FTIR)

Fourier-transform infrared spectroscopy (FTIR) was used to characterize the protein and lipid content of REVs based on the specific vibrations of these molecules^24,31^. FTIR measurements were carried out using a Varian 2000 spectrometer (Scimitar Series, USA), fitted with a diamond attenuated total reflection cell (‘Golden Gate’ single reflection ATR unit, Specac, United Kingdom). Approximately 5 μl of the sample was pipetted onto the diamond ATR surface and a thin dry film was obtained by slowly evaporating the solvent under ambient conditions (approx. 10 min). Typically, 64 scans were collected at a nominal resolution of 2 cm^−1^. ATR correction, buffer background spectral subtraction and other spectral evaluations were performed with the Grams/32 software package (Galactic Inc., USA).

### Fluorescence size exclusion chromatography (Flu-SEC)

Anti-CD235a (anti - Glycophorin A) conjugated with phycoerythrin (PE-CD235a, BioLegend, USA) and wheat germ agglutinin conjugated with Alexa647 fluorochrome (Alexa647-WGA, Thermo Fisher Scientific, USA) were used to label REVs. 100 µL REV sample was incubated with PE-CD235a at 0.8 to 8 μg/mL concentration or with Alexa647-WGA at 0.2 to 2 μg/mL concentration for 30 min at 37 °C. 10 µL of labeled REV sample was injected into a Jasco HPLC system (Jasco, Tokyo, Japan) consisting of a PU-2089 pump with an FP-2020 fluorescence detector controlled by the Chromnav software v. 1.17.02. Tricorn 5/200 glass columns (GE Healthcare Bio-Sciences AB, Sweden), filled with Sepharose CL-2B were used, and the eluent was PBS with a flow rate of 0.5 mL/min. The fluorescence chromatograms were collected at excitation and emission wavelength corresponding to PE (565/578 nm) and Alexa647 (650/665 nm) fluorochromes.
